# *Heteroonops* (Araneae, Oonopidae) spiders from Hispaniola: the discovery of ten new species

**DOI:** 10.3897/zookeys.964.51554

**Published:** 2020-08-27

**Authors:** Nadine Dupérré, Charlotte Francisco, Ella Santana-Propper, Ingi Agnarsson, Greta J. Binford

**Affiliations:** 1 Technical Assistant, Department of Arachnology, Centrum für Naturkunde, Universität de Hamburg, Germany Universität de Hamburg Hamburg Germany; 2 Lewis & Clark College, 0615 SW Palatine Hill Rd. Portland, Oregon, 97219, USA Lewis & Clark College Portland United States of America; 3 University of Vermont, Department of Biology, 109 Carrigan Drive, Burlington, VT, 05405-0086, USA University of Vermont Burlington United States of America; 4 Department of Entomology, National Museum of Natural History, Smithsonian Institution, Washington, DC, USA National Museum of Natural History Washington United States of America

**Keywords:** biodiversity hotspot, Caribbean biogeography, Goblin spiders, molecular phylogeny

## Abstract

The Caribbean biodiversity hotspot harbors vast reserves of undiscovered species. A large-scale inventory of Caribbean arachnids (CarBio) is uncovering new species across the arachnid tree of life, and allowing inference of the evolutionary history that has generated this diversity. Herein we describe ten new species of *Heteroonops* (Oonopidae, or goblin spiders), from Hispaniola: *H. scapula***sp. nov.**, *H. jurassicus***sp. nov.**, *H. aylinalegreae***sp. nov.**, *H. verruca***sp. nov.**, *H. renebarbai***sp. nov.**, *H. yuma***sp. nov.**, *H. carlosviquezi***sp. nov.**, *H. gabrielsantosi***sp. nov.**, *H. solanllycarreroae***sp. nov.** and *H. constanza***sp. nov.** The occurrence of the pantropical type species *Heteroonops spinimanus* (Simon, 1891) is reported and new localities are given for: *H. validus* (Bryant, 1948), *H. vega* (Platnick & Dupérré, 2009) and *H. castelloides* (Platnick & Dupérré, 2009). Molecular phylogenies indicate substantial genetic divergence separating these taxa. This work adds to evidence that the depth of diversity in the Caribbean biodiversity hotspot is particularly striking for tiny taxa living in leaf litter.

## Introduction

The Greater Antilles islands form the most species-rich landmasses in the Caribbean biodiversity hotspot. These islands serve as exceptional systems for studies of species formation and biogeography (Ricklefs and Bermingham 2008). Our ongoing large-scale inventory of Caribbean arachnids (CarBio) is rapidly uncovering new species across the arachnid tree of life and offering new insight into Caribbean biogeography (e.g., Dziki et al. 2015; Agnarsson et al. 2018; Chamberland et al. 2018; Čandek et al. 2019; Tong et al. 2019; Čandek et al. 2020). Yet the biodiversity of many of these islands, including Hispaniola, remains poorly known, especially with respect to tiny cryptic arthropods, such as oonopid spiders. The family Oonopidae currently includes 1846 species distributed in 113 genera, making it the 8^th^ largest spider family (World Spider Catalog 2020). In 2006, the Planetary Biodiversity Inventory (PBI, 2020) project on Oonopidae was launched. At the time only 459 species of Oonopidae were known (PBI, 2020). In eleven years, the PBI project led to the discovery and descriptions of nearly 1300 new oonopid species, increasing our knowledge of the fauna by 300%. Yet, new species continue to be discovered as new areas are more thoroughly sampled, such as during the ongoing Caribbean arachnid biodiversity inventory (project CarBio).

Oonopidae are small (1.0–5.0 mm) yellow, orange to bright red haplogyne spiders. Most members of this family are found living in leaf litter, but some live in canopies (Fannes et al. 2008, Platnick and Dupérré 2011b) or caves (Chamberlin and Ivie 1938), and some are termite nest inquilines (Benoit 1964) or even ant-mimics (Fannes and Jocqué 2008; Platnick and Dupérré 2011b). Oonopids typically have six large contiguous eyes (Ubick 2005), but some species have only two (Platnick 2000), or lack eyes altogether (Chamberlin and Ivie 1938; Benoit 1964; Baehr and Ubick 2010). Oonopids show other striking morphological features, including some with elongated carapace prongs (Abrahim et al. 2012), clypeal prongs (Platnick and Dupérré 2011a) and various cheliceral and endite modifications (e.g., Kranz-Baltensperger 2012; Tong et al. 2018). But an even more peculiar morphological feature is the occurrence of male palpal asymmetry, extremely rare in spiders (Huber et al. 2007), but found in oonopid genera such as *Escaphiella*, *Paradysderina* (Platnick and Dupérré 2009, 2011c). In *Paradysderina* the left and right male palps are so different that if observed independently, even experienced taxonomists would consider them to belong to distinct species (Platnick and Dupérré 2011c).

Platnick and Dupérré (2009) revised the genus *Heteroonops*, including 14 species, of which 10 were new. The type species of the genus, *Heteroonops spinimanus* (Simon, 1892), is pantropical, while the remainder of the group has a circum-Caribbean distribution, occurring from Mexico to Dominica (Platnick and Dupérré 2009). In 2009, four species were known to occur in Dominican Republic: *Heteroonops castelloides* (Platnick & Dupérré, 2009), *H. iviei* (Platnick & Dupérré, 2009), *H. validus* (Bryant, 1948) and *H. vega* (Platnick & Dupérré, 2009). Here we describe ten new species and report for the first time the presence of the pantropical genotype, *H. spinimanus*, as well as new localities for *H. vega*, *H. castelloides* and *H. validus*. We demonstrate substantial genetic divergence between these species and analyze biogeographic patterns within Hispaniola using mitochondrial phylogenies.

## Material and methods

### Collections examined

All 66 specimens examined are from the 2012 CarBio expedition to Dominican Republic, unless otherwise noted. They were all found in leaf litter samples that were sifted in the field and either hand sorted, or extracted through Berlese funnels. Specimens are stored at the Natural History Museum in Vermont, USA (UVM); type specimens are deposited at the National Museum of Natural History, Smithsonian Institution, Washington, USA (NMNH, USNMENT). Specimens were roughly sorted in-field and stored in 95% ethanol at -20 °C upon return to the laboratory. Species determination was done through morphological assessment, followed by molecular phylogenetic analyses. Genetic divergences guided further morphological assessment and final species delineation.

### Morphological assessment

Specimens were collected and examined in 95% ethanol under a SMZ-U Nikon dissection microscope. A Nikon Coolpix 950 digital camera attached to the microscope was used to photograph all the structures to be illustrated. The digital photos were used to trace proportions and the illustrations were detailed and shaded by referring back to the structure under the microscope. Female genitalia were excised using a sharp entomological needle and submerged in lactic acid to clear internal structures. The structures were photographed and illustrated as explained above. All measurements are in millimeters. For complete morphological description of the genus see Platnick and Dupérré (2009: 17–21). Nomenclatural morphology follows Platnick and Dupérré (2009).

### Molecular analyses

DNA extraction was done with the QIAGEN DNeasy Tissue Kit (Qiagen, Inc., Valencia, CA). We sequenced fragments of the mitochondrial Cytochrome c oxidase subunit 1 (COI) and 16S ribosomal RNA (16S), which are typically effective phylogenetic markers at low taxonomic levels for spiders. We amplified COI with LCO1490-2776 and 16S with 16SF and 16SR using standard protocols (see e.g., Agnarsson et al. 2007). PCR products were sequenced at the University of Arizona, Beckman Genomics, or the Smithsonian Institution. Sequences were interpreted from chromatograms using Phred and Phrap (Green and Ewing 2002, Green 2009) within the Chromaseq module (Maddison and Maddison 2020) in Mesquite 3.61 (Maddison and Maddison 2019), with default parameters. The sequences were then proofread by examining chromatograms by eye.

The taxon sampling in our final dataset included mitochondrial sequences for 37 of 38 *Heteroonops* from the Dominican Republic in our dataset (Table 1). We obtained COI data for all 37 of these specimens, and 16S for 32 of 37. Neither CO1 nor 16S amplified from the single representative of *H. solanllycarreroae* sp. nov. The concatenated alignment is 1114 nucleotides.

**Table 1.** 

Species (ND 17)	sex	type?	Locality	Latitude / Longitude	elev m	CO1	16s	GenBank Label	Specimen Name
*H. spinimanus*	f		DR Beach trail to Cueva del Puente, Parque Nacional del Este	18.32902N, 068.80995W	0	MT636140	MT635438	H._spinimanus_f	H. spin 01-1
*H. verruca* sp. nov.	m	holotype	DR Cachote Biosphere Reserve	18.09786N, 071.18925W	1200	MT636136	MT635434	H._verruca_n_sp_m1	H. verr 37-1
	f	paratype	DR Cachote Biosphere Reserve	18.09786N, 071.18925W	1200	MT636137	MT635435	H._verruca_n_sp_f1	H. verr 37-2
	m		DR Cachote Biosphere Reserve	18.09786N, 071.18925W	1200	MT636139	MT635437	H._verruca_n_sp_m2	H. verr 37-3
	m		DR Cachote Biosphere Reserve	18.09786N, 071.18925W	1200	MT636138	MT635436	H._verruca_n_sp_m3	H. verr 37-4
*H. validus*	m		DR Inside cueva del puente, Parque Nacional del Este	18.3816N, 068.8017W	25	MT636112	MT635415	H._validus_m1	H. val 02-1
	f		DR Inside cueva del puente, Parque Nacional del Este	18.3816N, 068.8017W	25	MT636113		H._validus_f1	H. val 02-2
	f		DR Inside cueva del puente, Parque Nacional del Este	18.3816N, 068.8017W	25	MT636114	MT635416	H._validus_f2	H. val 02-3
	m		DR Inside cueva del puente, Parque Nacional del Este	18.3816N, 068.8017W	25	MT636115		H._validus_m2	H. val 02-4
	m		DR Inside cueva del puente, Parque Nacional del Este	18.3816N, 068.8017W	25	MT636116		H._validus_m3	H. val 02-5
*H. carlosviquezi* sp. nov.	f	holotype	DR Loma Quita Espuela	19.34405N, 069.46635W	200	MT636111	MT635414	H._carlosviquezi_n_sp_f	7B11-2
*H. castelloides*	m		DR Loma Quita Espuela	19.34405N, 069.46635W	200	MT636124	MT635423	H._castelloides_m	H. cast 11-1
*H. vega*	m		DR Loma Quita Espuela	19.34405N, 069.46635W	200	MT636123		H._vega_m	H. veg 11-3
*H. yuma* sp. nov.	f	holotype	DR Loma Quita Espuela	19.34405N, 069.46635W	200	MT636122	MT635422	H._yuma_n_sp_f1	H. veg 11-1
	f	paratype	DR Loma Quita Espuela	19.34405N, 069.46635W	200	MT636121	MT635421	H._yuma_n_sp_f2	H. veg 11-2
*H. aylinalegreae* sp. nov.	m		DR Los Haitises: Cueva la Arena	19.08013N, 069.4649W	17	MT636132	MT635430	H._aylinalegreae_n_sp_m3	H. five 07-1
*H. renebarbai* sp. nov.	m	holotype	DR Los Haitises: Cueva la Arena	19.08013N, 069.4649W	17	MT636110	MT635413	H._renebarbai_n_sp_m	H. six 07-1
*H. aylinalegreae* sp. nov.	m	holotype	DR Parque del Este	18.355536N, 068.61825W	46	MT636128	MT635427	H._aylinalegreae_n_sp_m1	H. five 03-1
	f		DR Parque del Este	18.355536N, 068.61825W	46		MT645158	H._aylinalegreae_n_sp_f3	H. five 03-2
	f		DR Parque del Este	18.355536N, 068.61825W	46	MT636131	MT635429	H._aylinalegreae_n_sp_f1	H. five 03-3
	f		DR Parque del Este	18.355536N, 068.61825W	46	MT636129		H._aylinalegreae_n_sp_f2	H. five 03-4
	m		DR Parque del Este	18.355536N, 068.61825W	46	MT636130	MT635428	H._aylinalegreae_n_sp_m2	H. five 03-5
*H. constanza* sp. nov.	m	holotype	DR Valle Nuevo (Jurassic Park)	18.688N, 070.596W	2100	MT636125	MT635424	H._constanza_n_sp_m	H. cast 24-1
	f	paratype	DR Valle Nuevo (Jurassic Park)	18.688N, 070.596W	2100	MT636126	MT635425	H._constanza_n_sp_f1	H. cast 24-2
	f	paratype	DR Valle Nuevo (Jurassic Park)	18.688N, 070.596W	2100	MT636127	MT635426	H._constanza_n_sp_f2	H. cast 24-3
*H. gabrielsantosi* sp. nov.	f	paratype	DR Valle Nuevo (Jurassic Park)	18.688N, 070.596W	2100	MT636133	MT635431	H._gabrielsantosi_n_sp_f2	H. one 24-1
	f	holotype	DR Valle Nuevo (Jurassic Park)	18.688N, 070.596W	2100	MT636135	MT635433	H._gabrielsantosi_n_sp_f1	H. one 24-2
	f	paratype	DR Valle Nuevo (Jurassic Park)	18.688N, 070.596W	2100	MT636134	MT635432	H._gabrielsantosi_n_sp_f3	H. one 24-3
*H. jurassicus* sp. nov.	m		DR Valle Nuevo (Jurassic Park)	18.688N, 070.596W	2100	MT636117	MT635417	H._jurassicus_n_sp_m1	H. jur 24-1
	m		DR Valle Nuevo (Jurassic Park)	18.688N, 070.596W	2100	MT636120	MT635420	H._jurassicus_n_sp_m2	H. jur 24-3
	f		DR Valle Nuevo (Jurassic Park)	18.688N, 070.596W	2100	MT636118	MT635418	H._ jurassicus_n_sp_f1	H. jur 24-4
	f		DR Valle Nuevo (Jurassic Park)	18.688N, 070.596W	2100	MT636119	MT635419	H._ jurassicus_n_sp_f2	H. jur 24-5
*H. scapula* sp. nov.	f	paratype	DR Valle Nuevo Rd	18.84633N, 070.74064W	2983	MT636109	MT635412	H._ scapula_n_sp_f	H. two 22-2
	m	holotype	DR Valle Nuevo, NP; Valle Nuevo Rd	18.84633N, 070.74064W	2983	MT636108		H._scapula_n_sp_m	H. two 22-1
*Oonopidae* sp 1	f		DR Los Haitises: Cueva la Arena	19.08013N, 069.4649W	17	MT636142	MT635440	Oonopidae_sp_1_DR_f	H. six 07-2
*Oonopidae* sp 2	f		PR Mona Island: Bajuga Empalme			MT636141	MT635439	Oonopidae_sp_2_Mona_f	H. mona 1
00392858 *Stenoonops portoricensis*	f		PR Ranger Station, Guanica Dry Forest	17.971472N, 066.86795W	154	MT636143		00392858_S._portoricensis	

For phylogenetic analyses, alignments were done in MAFFT (Katoh 2013) through the online portal EMBL-EBI, using default settings but increasing the tree rebuilding and maxiterate settings to 100. Gaps were treated as missing characters. The aligned sequences for COI, and 16S, were tested for the best fitting substitution model using the program Jmodeltest 2.1.7 (Darriba et al. 2012). The best models for each gene, among the 24 models available in MrBayes, were GTR+G for 16S and GTR+I+G for COI. We conducted Bayesian analyses using MrBayes V3.2.3 through the online portal CIPRES (Miller et al. 2010) on the concatenated mtDNA dataset. The Bayesian analyses ran 10,000,000 generations, sampling every 1000 generations. We used Tracer (Drummond and Rambaut 2007) to verify proper convergence of runs and sufficient sampling of priors.

### Abbreviations

**Somatic morphology**

ALE anterior lateral eye

PLE posterior lateral eye

PME posterior median eye

**Genitalia (female)**

ar anterior receptaculum

ef epigastric furrow

es epigastric scutum

pr posterior receptaculum

ps postepigastric scutum

wp wing like projections

**Genitalia (male)**

c bulb

c conductor

e embolus

## Results

**Figure 1.**
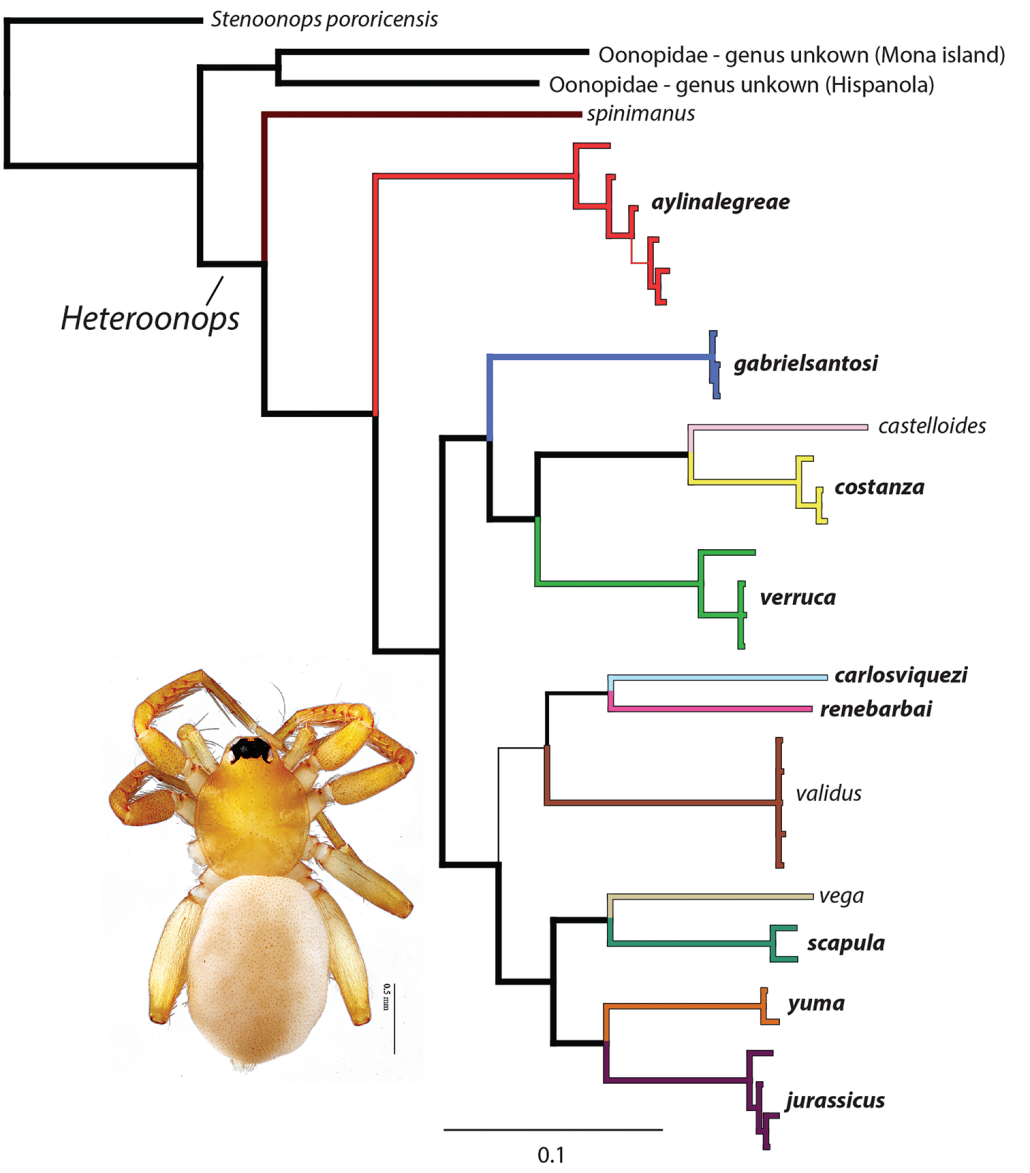
Summary phylogeny of the included species rendering support for the monophyly (multiple samples per species) or exclusivity (single specimens) of each species dealt with here. Species color scheme equals that on map in Figure 40. New species are highlighted in **bold**. Thick branches have >95% posterior probability support, thin branches have >75% posterior probability support. Scale bar indicates the number of expected changes on branches. Inset photo of female *H. jurassicus* sp. nov. For more detailed specimen-level phylogeny see Suppl. material 1.

The ten new species of *Heteroonops* presented in this work are genetically distinct and distinguishable morphologically. They were all collected in leaf litter samples from forest or cave habitats in Hispaniola ranging from near sea level to 2983 m. Mitochondrial genetic divergences and patterns of relationships belie a deep and old history of *Heteroonops* on Hispaniola (Fig. 1).

### Taxonomy

#### Oonopidae

##### Heteroonops

Taxon classificationAnimaliaAraneaeOonopidae

Dalmas, 1916

48E80FC4-CD7C-5FA5-B6EA-FA1ABA5B4ABA

###### Composition.

*H. andros* Platnick & Dupérré, 2009, *H. aylinalegreae* sp. nov., *H. carlosviquezi* sp. nov., *H. castelloides* Platnick & Dupérré, 2009, *H. castellus* (Chickering, 1971), *H. colombi* Dumitrescu & Georgescu, 1983, *H. constanza* sp. nov., *H. croix* Platnick & Dupérré, 2009, *H. gabrielsantosi* sp. nov., *H. iviei* Platnick & Dupérré, 2009, *H. jurassicus* n. sp, *H. macaque* Platnick & Dupérré, 2009, *H. murphyorum* Platnick & Dupérré, 2009, *H. renebarbai* sp. nov., *H. saba* Platnick & Dupérré, 2009, *H. scapula* sp. nov., *H. singulus* (Gertsch & Davis, 1942), *H. solanllycarreroae* sp. nov., *H. spinigata* Platnick & Dupérré, 2009, *H. spinimanus* (Simon, 1891), *H. toro* Platnick & Dupérré, 2009, *H. validus* (Bryant, 1948), *H. vega* Platnick & Dupérré, 2009, *H. verruca* sp. nov., *H. yuma* sp. nov.

###### Distribution.

Mexico, Costa Rica, Bahama Islands, Cuba, Jamaica, Dominican Republic, Puerto Rico, Virgin Islands, Saba, Montserrat and Dominica (*H. spinimanus* (Simon, 1891) presents a pantropical distribution).

###### Diagnosis.

Males are easily diagnosed from all other Oonopidae by the presence of one or two backward-pointing projections on the male palpal endites (Figs 29–33). Females are easily diagnosed by their elongated, spinose pedipalpi (Platnick and Dupérré 2009, fig. 181).

##### Heteroonops scapula

Taxon classificationAnimaliaAraneaeOonopidae

Dupérré sp. nov.

9AA74717-8748-5B35-B4B5-F32542B34701

http://zoobank.org/00009E22-3BB0-462B-855D-E4B136FEDCB2

Figs 2–5 , 34 , 40

###### Type material.

Male holotype from Dominican Republic, La Vega Province, Constanza, Valle Nuevo National Park, 18.84633N, 70.74064W, 2983 m, 26.vi.2012, team CarBio (NMNH, USNMENT 01747000). One female paratype, same data.

###### Etymology.

The specific epithet is a noun in apposition meaning wings, in reference to the large wing-like structures of the female internal genitalia.

###### Diagnosis.

**Figures 2–5.**
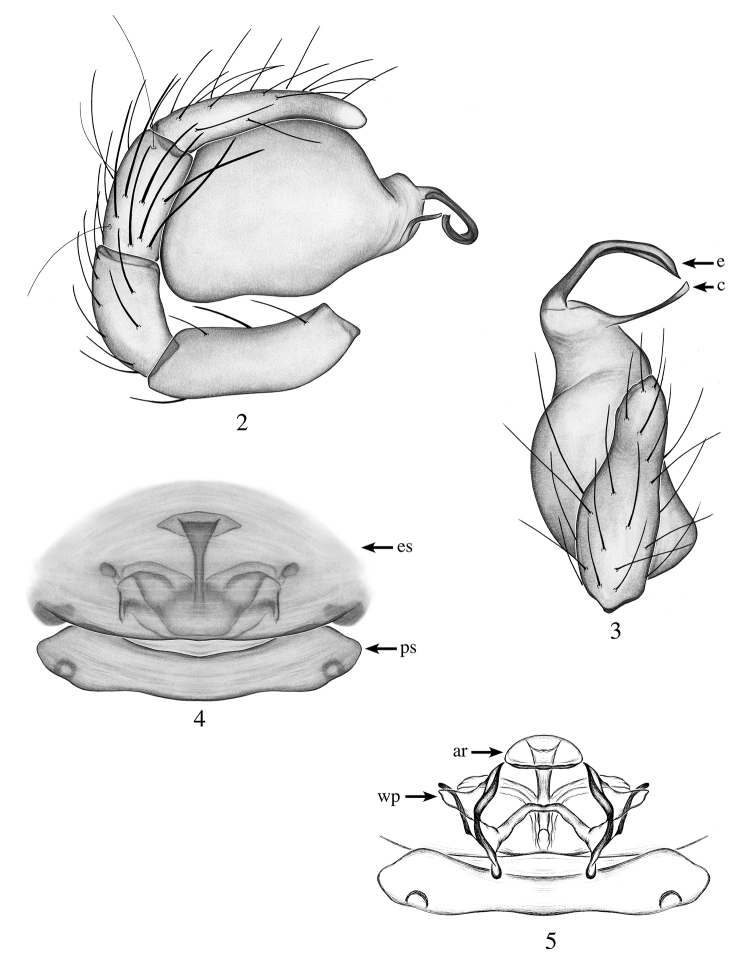
*Heteroonops scapula* sp. nov. Male (**2, 3**), female (**4, 5**). **2** Palp, prolateral view **3** palp, apical view **4** epigynal region, ventral view **5** internal genitalia dorsal view.

Males are diagnosed from all species by the combination of the following characters: constricted tip of palpal bulb and their bent embolus, wider apically, long conductor reaching the tip of the embolus (Figs 2, 3); females are diagnosed by the large, anterior wing-like projections of their internal genitalia and triangular anterior receptaculum (Fig. 5).

###### Description.

**Male (holotype)**: Total length: 1.9; carapace length: 1.0; carapace width: 0.7. ***Cephalothorax***: Carapace ovoid; shiny, bright orange; pars cephalica flat. Sternum yellow; longer than wide; covered entirely with long dark setae. Endites yellow with one elongated and thin apical backward-pointing projection (Fig. 34); labium light yellow. Clypeus vertical; short (1/2× radius of ALE). Chelicerae yellow; promargin and retromargin without teeth; fangs normal 1/3 length of chelicerae. ***Eyes***: Six eyes surrounded by black pigmentation; ALE largest, oval, PME squared; PLE smallest, oval; ALE separated by their radius; ALE-PLE touching; PLE-PME touching; PME touching. ***Abdomen***: Oval; light gray covered dorsally with long dark setae; epigastric and postepigastric scuta light orange, well sclerotized. ***Legs***: Yellow; tibia I with five pairs of ventral spines, metatarsus I with 2 pairs of ventral spines; leg formula undetermined, missing legs II-III-IV. ***Genitalia***: Palpal segments light yellow; palpal bulb whitish. Palpal femur, patella and tibia with spines prolaterally (Fig. 2). Palpal bulb ovoid constricted at tip (Fig. 2); embolus long, bent medially, wider apically; conductor elongated and thin, wider apically, reaching the tip of the embolus (Fig. 3).

**Female (paratype)**: Total length: 1.98; carapace length: 0.94; carapace width: 0.74. ***Cephalothorax***: Carapace ovoid; shiny, bright orange; pars cephalica flat. Sternum, labium and chelicerae: as in male. Endites without projection. ***Eyes***: Same as male. ***Abdomen***: Oval; gray; epigastric and postepigastric scuta orange, well sclerotized (Fig. 4). ***Legs***: Color as in male; all legs missing; all palpal segments with strong spines. ***Genitalia***: Epigynal region not protruding, with large structure visible through the epigastric scutum (Fig. 4). Internal genitalia with triangular anterior receptaculum, projecting posteriorly into a plate-like extrusion; posterior receptaculum not observed; wing-like projections well sclerotized, tridimensional (Fig. 5).

###### Other material examined.

None.

###### Distribution.

Dominican Republic, La Vega Province (Fig. 40).

##### Heteroonops jurassicus

Taxon classificationAnimaliaAraneaeOonopidae

Dupérré sp. nov.

CF69ED80-E608-58EF-8E6D-A815545DF104

http://zoobank.org/F8D0A1A4-B6CF-438F-BADD-7C5FCAAA995B

Figs 6–9 , 35 , 40

###### Type material.

Male holotype from Dominican Republic, La Vega Province, Constanza, Valle Nuevo National Park, ‘Jurassic Park’, 18.688N, 70.596W, 2100 m, 26.vi.2012, team CarBio (NMNH, USNMENT 01747001). Two female paratypes, same data.

###### Etymology.

The specific epithet is a noun in apposition taken from the type locality, Jurassic Park, Dominican Republic.

###### Diagnosis.

Males are distinguished from all species of the genera by the spatula-shaped tip of the embolus (Fig. 7). Females are distinguished by their large funnel-shaped anterior receptaculum (Fig. 9).

###### Description.

**Figures 6–9.**
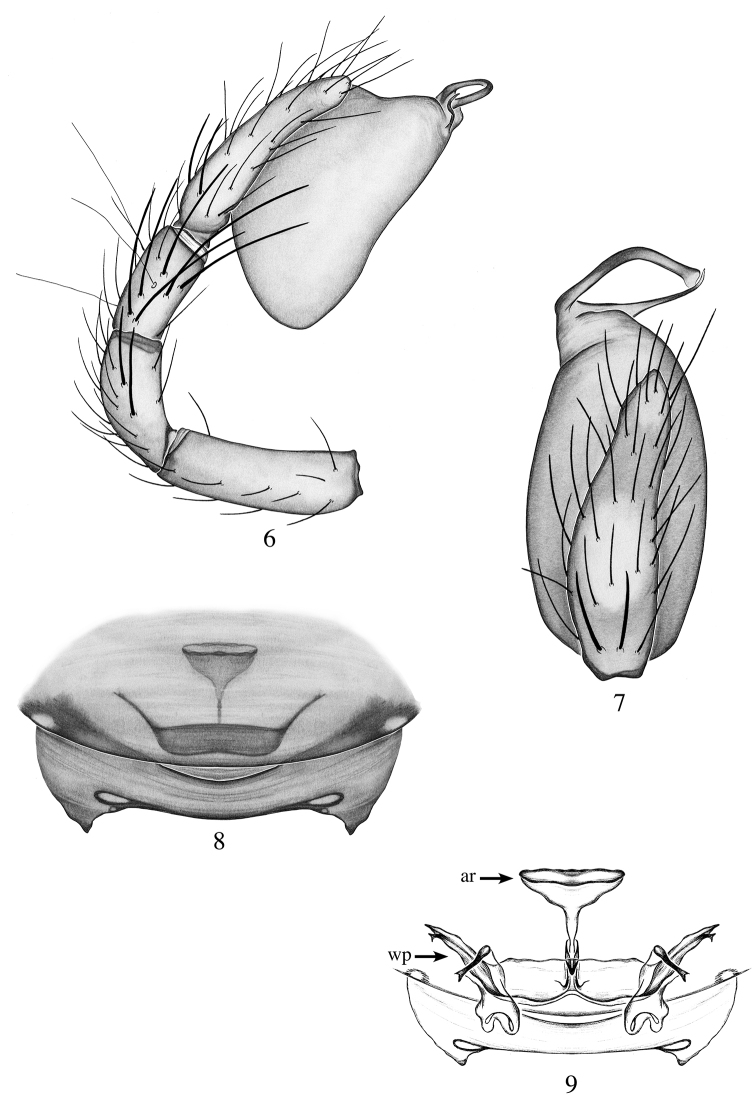
*Heteroonops jurassicus* sp. nov. Male (**6, 7**), female (**8, 9**). **6** Palp, prolateral view **7** palp, apical view **8** epigynal region, ventral view **9** internal genitalia, dorsal view.

**Male (holotype)**: Total length: 1.93; carapace length: 1.03; carapace width: 0.96. ***Cephalothorax***: Carapace ovoid; shiny, bright orange; pars cephalica slightly elevated. Sternum orange; longer than wide; covered entirely with long dark setae. Endites orange with one very small apical backward-pointing projection (Fig. 35); labium light orange. Clypeus vertical; short (1/2× radius of ALE). Chelicerae orange; promargin and retromargin without teeth; fangs long, 2/3 the length of the chelicerae. ***Eyes***: Six eyes surrounded by black pigmentation; ALE largest, oval; PME rectangular; PLE smallest, oval; ALE separated by their radius; ALE-PLE touching; PLE-PME touching; PME touching. ***Abdomen***: Oval; beige dorsally covered with long dark setae; epigastric and postepigastric scuta orange, well sclerotized. ***Legs***: Orange; tibia I with five pairs of ventral spines, metatarsus I with two pairs of ventral spines; leg formula 4123. ***Genitalia***: Palpal segments yellow; palpal bulb whitish. Palpal patella, tibia and tarsus with spines prolaterally (Fig. 6). Palpal bulb ovoid slightly constricted at tip (Fig. 6); embolus long, bent medially with transparent spatula-shaped tip; conductor long and thin reaching the tip of the embolus (Fig. 7).

**Female (paratype)**: Total length: 2.12; carapace length: 0.92; carapace width: 0.76. ***Cephalothorax***: Carapace ovoid; shiny, yellow; pars cephalica flat. Sternum and labium light yellow. Chelicerae and endites light yellow, not modified. ***Eyes***: as in male. ***Abdomen***: Oval, light beige; epigastric and postepigastric scuta orange, well sclerotized (Fig. 8). ***Legs***: Light yellow; tibia I with five pairs of ventral spines, metatarsus I with two pairs of ventral spines; leg formula 4123; all palpal segments with strong spines. ***Genitalia***: Epigynal region not protruding, with funnel-shaped and rectangular structures visible through the epigastric scutum (Fig. 8). Internal genitalia with funnel-shaped anterior receptaculum; posterior receptaculum not observed; wing-like projections well sclerotized, tridimensional (Fig. 9)

###### Other material examined.

Same data as type specimens: 1♂ (USNMENT 00788060), 1♂ (USNMENT 00788048), 1♀ (USNMENT 00788084); 3♂, 4♀ (UVM).

###### Distribution.

Dominican Republic, La Vega Province (Fig. 40).

##### Heteroonops aylinalegreae

Taxon classificationAnimaliaAraneaeOonopidae

Dupérré sp. nov.

92425C20-8C32-56DB-A678-BAE3CC81B417

http://zoobank.org/EBB74055-FC21-4252-AD4C-F4628928F811

**Figures 10–13.**
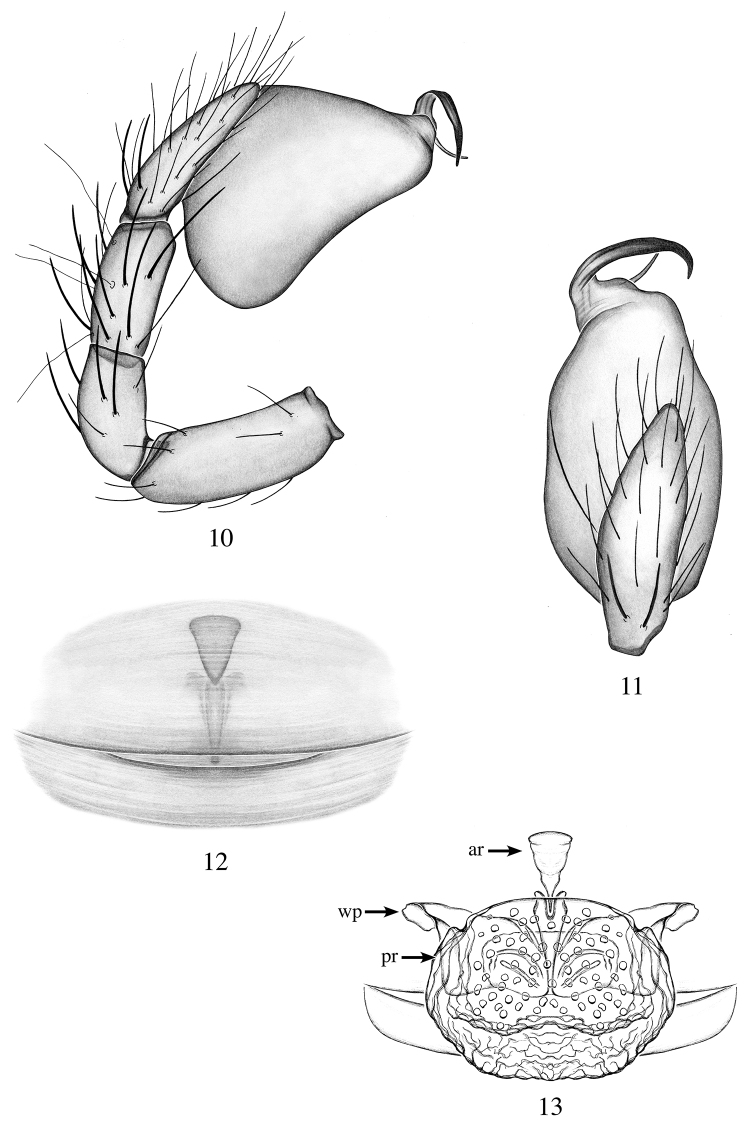
*Heteroonops aylinalegreae* sp. nov. Male (**10, 11**), female (**12, 13**). **10** Palp, prolateral view **11** palp, apical view **12** epigynal region, ventral view **13** internal genitalia, dorsal view.

Figs 10–13 , 36 , 40

###### Type material.

Male holotype from Dominican Republic, La Alta Gracia Province, Occidental, San Rafael, del Este National Park, 18.355536N, 68.6182518W, 46 m, 7–8.vi.2012, team CarBio (NMNH, USNMENT 01747002). One male and four female paratypes, same data (USNMENT 01747003).

###### Etymology.

The specific epithet is a noun in apposition honoring local arachnologist and CarBio collaborator Aylin Alegre.

###### Diagnosis.

Males are diagnosed from all *Heteroonops* by the combination of the following characters: embolus well sclerotized, not spatulated apically; short conductor not reaching the tip of the embolus (Fig. 11); females are diagnosed by their inverse triangular anterior receptaculum and large posterior receptaculum (Fig. 13).

###### Description.

**Male (holotype)**: Total length: 1.65; carapace length: 0.79; carapace width: 0.67. ***Cephalothorax***: Carapace ovoid; shiny, light yellow; pars cephalica flat. Sternum light yellow; longer than wide; covered entirely with long dark setae. Endites light yellow with one small apical backward-pointing projection (Fig. 35); labium light yellow. Clypeus vertical; short (1/2× radius of ALE). Chelicerae yellow; promargin and retromargin without teeth; fangs normal, 1/3 length of chelicerae. ***Eyes***: Six eyes surrounded by black pigmentation; ALE largest, oval; PME squared; PLE smallest, oval; ALE separated by their radius; ALE-PLE touching; PLE-PME touching; PME touching. ***Abdomen***: Oval; light gray, dorsally covered with long dark setae; epigastric and postepigastric scuta light yellow, not well sclerotized. ***Legs***: Femora whitish; other legs segments light yellow; tibia I with one pair of ventral spines, metatarsus I with two pairs of ventral spines; leg formula 4123. ***Genitalia***: Palpal segments yellow; palpal bulb whitish. Palpal patella, tibia and tarsus with spines prolaterally (Fig. 10). Palpal bulb ovoid slightly constricted at tip (Fig. 10); embolus well sclerotized, curved with pointed tip; conductor short and pointed not reaching tip of the embolus (Fig. 11).

**Female (paratype)**: Total length: 1.89; carapace length: 0.81; carapace width: 0.67. ***Cephalothorax***: Carapace, sternum, labium and chelicerae: as in male. Endites without projection. ***Eyes***: Same as male. ***Abdomen***: Oval; light gray; epigastric and postepigastric light yellow, not well sclerotized (Fig. 12). ***Legs***: Color as in male; tibia I with three pairs of ventral spines, metatarsus I with two pairs of ventral spines; leg formula 4123; all palpal segments with strong spines. ***Genitalia***: Epigynal region not protruding, with tulip-shaped structure visible through the epigastric scutum (Fig. 12). Internal genitalia with inverted triangular anterior receptaculum; posterior receptaculum large, pouch-shaped, wrinkled with pore field; wing-like projections short (Fig. 13).

###### Other material examined.

1♂ Dominican Republic, Hato Mayor Province, Occidental, San Rafael de Yuma, Parque Nacional los Haitises, Cueva La Arena, 19.08013N 69.4649W, 17♂, 12.vi.2012, team CarBio (UVM); 1♂3♀ Dominican Republic, La Alta Gracia Province, Occidental, San Rafael, del Este National Park, 18.355536N, 68.6182518W, 46 m, 7–8.vi.2012, team CarBio (UVM).

###### Distribution.

Dominican Republic, La Alta Gracia and Hato Mayor provinces (Fig. 40).

##### Heteroonops verruca

Taxon classificationAnimaliaAraneaeOonopidae

Dupérré sp. nov.

DE2A5A9C-39DD-523D-A860-59AE4CE9A393

http://zoobank.org/18B6E9E1-0B6C-45C8-B724-85C0A3279651

**Figures 14–18.**
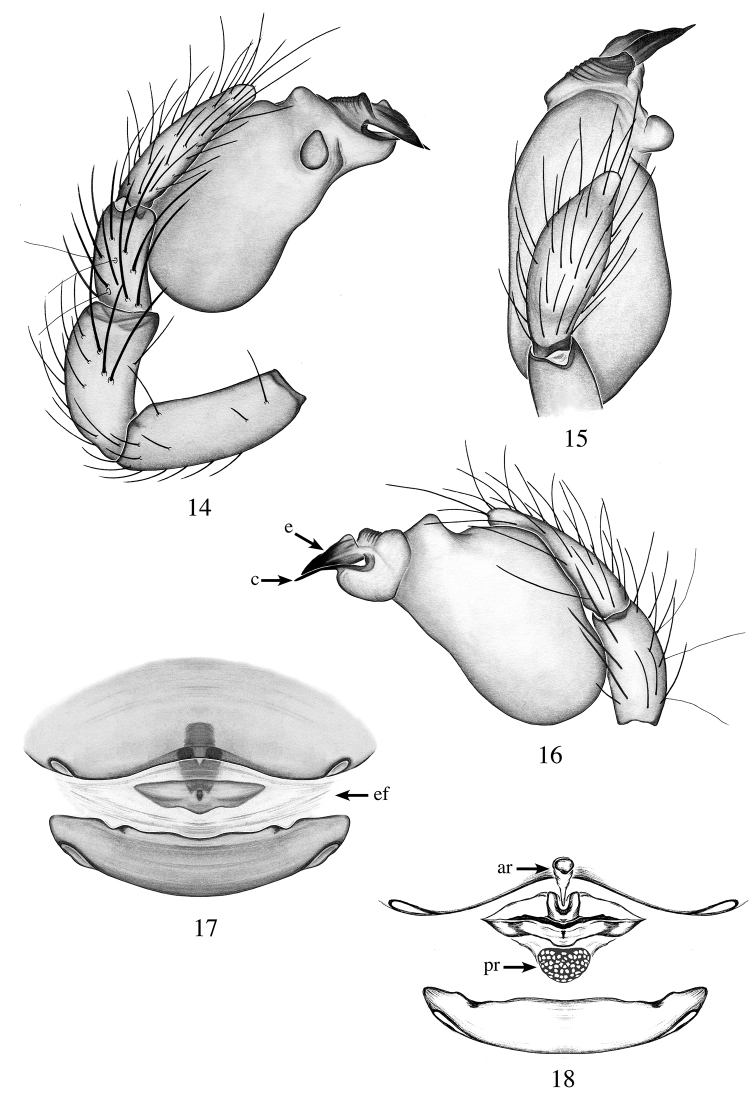
*Heteroonops verruca* sp. nov. Male (**14–16**), female (**17, 18**). **14** Palp, prolateral view **15** palp, apical view **16** palp, retrolateral view **17** epigynal region, ventral view **18** internal genitalia, dorsal view.

Figs 14–18 , 37 , 40

###### Type material.

Male holotype from Dominican Republic, Barahona Province, Cachote Biosphere Reserve, 18.09786N, 71.18925W, 1200 m, 7.vii.2012, team CarBio (NMNH, USNMENT 01747004). One female paratype, same data.

###### Etymology.

The specific epithet is a noun in apposition meaning wart in reference to the male palpal bulb bearing a wart-like projection.

###### Diagnosis.

Males can be diagnosed from all species by the wart-like projection on the prolateral side of the bulb (Fig. 14); females can be diagnosed by their small heart-shaped posterior receptaculum (Fig. 18).

###### Description.

**Male (holotype)**: Total length: 1.9; carapace length: 0.95; carapace width: 0.79. ***Cephalothorax***: Carapace ovoid; shiny, bright yellow; pars cephalica flat. Sternum yellow; longer than wide; covered entirely with long dark setae. Endites yellow with one large, median backward-pointing projection (Fig. 37); labium yellow. Clypeus vertical; short (1/2× radius of ALE). Chelicerae yellow; promargin and retromargin without teeth; fangs normal, 1/3 the length of the chelicerae. ***Eyes***: Six eyes surrounded by black pigmentation; ALE largest, oval; PME rectangular; PLE smallest, oval; ALE separated by their radius; ALE-PLE touching; PLE-PME touching; PME touching. ***Abdomen***: Oval; light beige covered dorsally with long dark setae; epigastric and postepigastric scuta light yellow, well sclerotized. ***Legs***: Femora with basal half whitish, apical half-light yellow, other legs segments light yellow; tibia I with three pairs of ventral spines, metatarsus I with two pairs of ventral spines; leg formula 4123. ***Genitalia***: Palpal segments yellow; palpal bulb whitish. Palpal patella and tibia with spines prolaterally (Fig. 14). Palpal bulb ovoid with apical triangular bump and prolateral wart-like projection (Figs 13, 14); embolus and conductor set on an oval base with apical ridges (Figs 15, 16); embolus well sclerotized, wide and triangular; conductor spine-like, well sclerotized reaching the tip of the embolus (Fig. 16).

**Female (paratype)**: Total length: 2.04; carapace length: 0.98; carapace width: 0.76. ***Cephalothorax***: Carapace, sternum, labium and chelicerae: as in male. Endites without projection. ***Eyes***: Same as male. ***Abdomen***: Oval, light beige; epigastric and postepigastric scuta orange, well sclerotized (Fig. 17). ***Legs***: Color as in male; leg I missing; all palpal segments with strong spines. ***Genitalia***: Epigynal region not protruding, with small, squared structure visible through the epigastric scutum, and triangular plate visible through the epigastric furrow (Fig. 17). Internal genitalia with triangular anterior receptaculum, projecting posteriorly; posterior receptaculum small, bulbous with pore field; wing-like projections not observed (Fig. 18).

###### Other material examined.

Same data as type specimens: 2♂ (UVM).

###### Distribution.

Dominican Republic, Barahona Province (Fig. 40).

##### Heteroonops renebarbai

Taxon classificationAnimaliaAraneaeOonopidae

Dupérré sp. nov.

7808B2FC-D64F-503F-BE41-15FB8458846C

http://zoobank.org/803999F5-7C2D-4CE6-9C83-6264977AA215

**Figures 19, 20.**
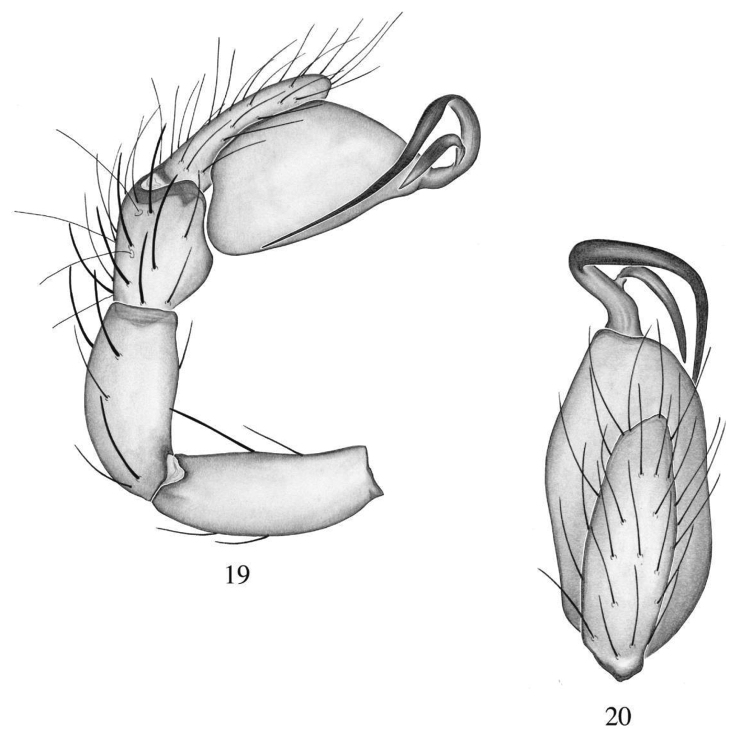
*Heteroonops renebarbai* sp. nov. Male. **19** Palp, prolateral view **20** palp, apical view.

Figs 19 , 20 , 38 , 40

###### Type material.

Male holotype from Dominican Republic, Hato Mayor Province, Occidental, San Rafael de Yuma, los Haitises National Park, outside Cueva La Arena, 19.08013N, 69.4649W, 17m, 12.vi.2012, team CarBio (NMNH, USNMENT 01747005).

###### Etymology.

The specific epithet is a noun in apposition honoring local arachnologist and CarBio collaborator René Barba.

###### Diagnosis.

Males are distinguished from most species by their elongated, thin embolus (Fig. 19); from *H. vega* by their long and pointed conductor (Fig. 20), flat and with denticles in the later (Platnick and Dupérré 2009, fig. 194).

###### Description.

**Male (holotype)**: Total length: 1.34; carapace length: 0.71; carapace width: 0.59. ***Cephalothorax***: Carapace ovoid; shiny, light yellow; pars cephalica flat. Sternum light yellow; longer than wide; covered entirely with long dark setae. Endites light yellow with an elongated apical backward-pointing projection with rounded tip (Fig. 38); labium light yellow. Clypeus vertical; short (1/2× radius of ALE). Chelicerae yellow; promargin and retromargin without teeth; fangs normal, 1/3 length of chelicerae. ***Eyes***: Six eyes surrounded by black pigmentation; ALE largest, oval; PME squared; PLE smallest, oval; ALE separated by their radius; ALE-PLE touching; PLE-PME touching; PME touching. ***Abdomen***: Oval; light beige covered dorsally with long dark setae; epigastric and postepigastric scuta light yellow, not well sclerotized. ***Legs***: Light yellow; tibia I with two pairs of ventral spines, metatarsus I with one pair of ventral spines; leg formula undertermined, legs II-III-IV missing. ***Genitalia***: Palpal segments light yellow; palpal bulb whitish. Palpal femur, patella and tibia with spines prolaterally (Fig. 19). Palpal bulb ovoid (Fig. 19); embolus well sclerotized, long and thin; conductor long and pointed, initiating at base of embolus (Figs 19, 20).

**Female**: Unknown.

###### Other material examined.

None.

###### Distribution.

Dominican Republic, Hato Mayor Province (Fig. 40).

##### Heteroonops yuma

Taxon classificationAnimaliaAraneaeOonopidae

Dupérré sp. nov.

2099344E-63AD-5A78-ACB4-B6F08F9C2142

http://zoobank.org/C9159DF2-1A78-4434-BA1E-65A51DD10D33

Figs 21 , 22 , 40

###### Type material.

Female holotype from Dominican Republic, Duarte Province, Occidental, San Rafael de Yuma, Loma Quita Espuela, 19.35504N, 70.111W, 200 m, 14.vi.2012, team CarBio (NMNH, USNMENT 01747006). Female paratype, same data (USNMENT 01747007).

###### Etymology.

The specific name is noun in apposition taken from the type locality, San Rafael de Yuma, Dominican Republic.

###### Diagnosis.

Females are distinguished from most species by the anterior receptaculum positioned on a narrow, short stalk; from *H. vega* by their larger anterior receptaculum projecting posteriorly (Fig. 22), not projecting in the later species (Platnick and Dupérré 2009, fig. 211).

###### Description.

**Figures 21, 22.**
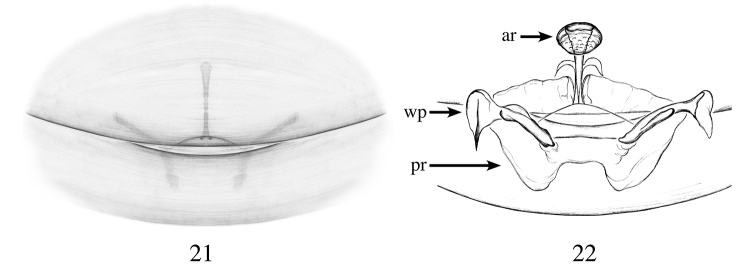
*Heteroonops yuma* sp. nov. Female. **21** Epigynal region, ventral view **22** internal genitalia, dorsal view.

**Female (holotype)** Total length: 1.86; carapace length: 0.76; carapace width: 0.61. ***Cephalothorax***: Carapace ovoid; shiny, whitish; pars cephalica flat. Sternum whitish; longer than wide; covered entirely with long dark setae. Endites withish, not modified; labium light whitish. Clypeus vertical; short (1/2× radius of ALE). Chelicerae pale yellow; promargin and retromargin without teeth; fangs normal, 1/3 length of chelicerae. ***Eyes***: Six eyes surrounded by black pigmentation; ALE largest, oval; PME squared; PLE smallest, oval; ALE separated by their radius; ALE-PLE touching; PLE-PME touching; PME touching. ***Abdomen***: Oval; yellowish; epigastric and postepigastric scuta pale yellow, not well sclerotized (Fig. 21). ***Legs***: whitish; tibia I with four pairs of ventral spines, metatarsus I with three pairs of ventral spines; leg formula 4123; all palpal segments with strong spines. ***Genitalia***: Epigynal region not protruding with faint structure visible through the scuta (Fig. 21). Internal genitalia with triangular anterior receptaculum, projecting posteriorly (Fig. 22); posterior receptaculum transparent, W-shaped; wing-like projections golf club-shaped (Fig. 22).

**Male**: Unknown.

###### Other material examined.

None.

###### Distribution.

Dominican Republic, Duarte Province (Fig. 40).

##### Heteroonops carlosviquezi

Taxon classificationAnimaliaAraneaeOonopidae

Dupérré sp. nov.

E89446B4-8D50-5470-9678-367D5BE422B5

http://zoobank.org/9192A67A-94FD-4CE5-852B-AE9586764724

Figs 23–25 , 40

###### Type material.

Female holotype from Dominican Republic, Duarte Province, Occidental, San Rafael de Yuma, Loma Quita Espuela, 19.35504N, 70.111W, 200 m, 14.vi.2012, team CarBio (NMNH, USNMENT 01747008).

###### Etymology.

The specific epithet is a noun in apposition honoring Costa Rican arachnologist and CarBio collaborator Carlos Viquez.

###### Diagnosis.

Females are easily diagnosed by their umbrella-shaped anterior receptaculum (Fig. 24).

###### Description.

**Figures 23–27.**
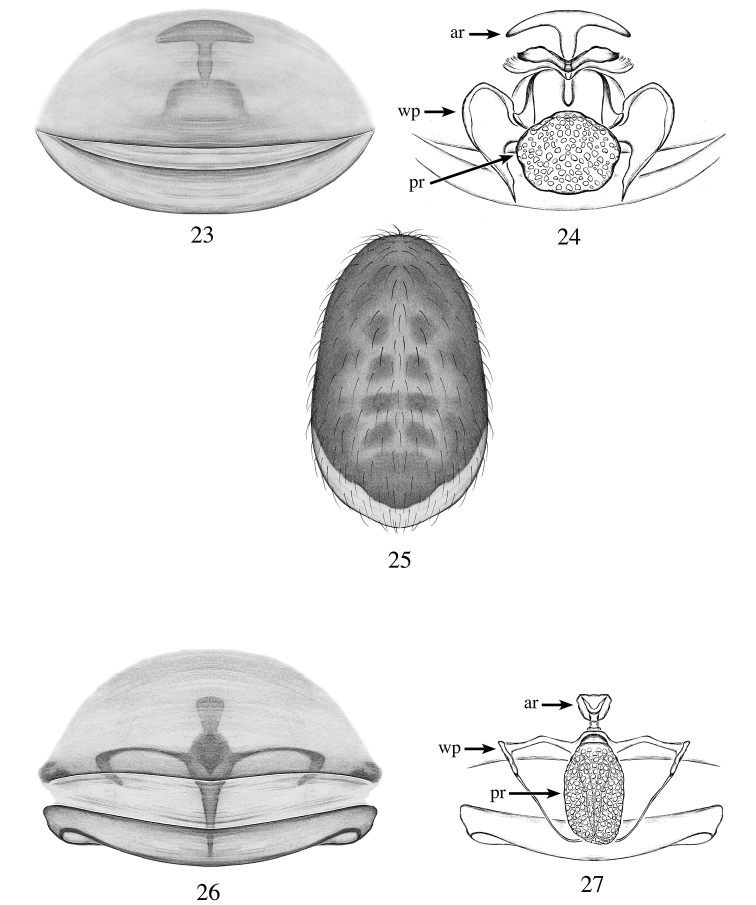
*Heteroonops carlosviquezi* sp. nov., female (**23–25**). *Heteroonops gabrielsantosi* sp. nov., female (**27**). **23** Epigynal region, ventral view **24** internal genitalia, dorsal view **25** abdomen, dorsal view **26** epigynal region, ventral view **27** internal genitalia, dorsal view.

**Female**: Total length: 2.06; carapace length: 0.96; carapace width: 0.8. ***Cephalothorax***: Carapace ovoid; shiny, light orange; pars cephalica flat. Sternum yellow; longer than wide; covered entirely with long dark setae. Endites yellow, not modified; labium light yellow. Clypeus vertical; short (1/2× radius of ALE). Chelicerae yellow; promargin and retromargin without teeth; fangs normal, 1/3 length of chelicerae. ***Eyes***: Six eyes surrounded by black pigmentation; ALE largest, oval; PME squared; PLE smallest, oval; ALE separated by their radius; ALE-PLE touching; PLE-PME touching; PME touching. ***Abdomen***: Oval; dark grayish-blue with pattern, apically whitish (Fig. 25); epigastric and postepigastric scuta light orange, well sclerotized (Fig. 23). ***Legs***: Orange; tibia I with four pairs of ventral spines, metatarsus I with three pairs of ventral spines; leg formula 4123; all palpal segments with strong spines. ***Genitalia***: Epigynal region not protruding, with bell-shaped structure visible through the epigastric scutum (Fig. 23). Internal genitalia with umbrella-shaped anterior receptaculum; posterior receptaculum globose with large pore field; wing-like projections large, ear-shaped (Fig. 24).

**Male**: Unknown.

###### Other material examined.

None.

###### Distribution.

Dominican Republic, Duarte Province (Fig. 40).

##### Heteroonops gabrielsantosi

Taxon classificationAnimaliaAraneaeOonopidae

Dupérré sp. nov.

8E94F832-848A-5967-B350-14A80DE0D75E

http://zoobank.org/33CC4CA3-3B84-43A9-978D-CF5391CEFEAC

Figs 26 , 27 , 40

###### Type material.

Female holotype from Dominican Republic, La Vega Province, Constanza, Valle Nuevo National Park, ‘Jurassic Park’, 18.688N, 70.596W, 2100 m, 26.vi.2012, team CarBio (NMNH, USNMENT 01747009). Two female paratypes (USNMENT 01747010, 01747011), same data.

###### Etymology.

The specific epithet is a noun in apposition honoring local arachnologist and CarBio collaborator Gabriel Santos.

###### Diagnosis.

Females can be diagnosed from all species by the arch wing-like projections of the internal genitalia and large oval posterior receptaculum (Fig. 27).

###### Description.

**Female**: Total length: 2.31; carapace length: 0.91; carapace width: 0.84. ***Cephalothorax***: Carapace ovoid; shiny, light yellow; pars cephalica flat. Sternum light yellow; longer than wide; covered entirely with long dark setae. Endites light yellow, not modified; labium light yellow. Clypeus vertical, short (1/2× radius of ALE). Chelicerae light yellow; promargin and retromargin without teeth; fangs normal, 1/3 length of chelicerae. ***Eyes***: Six eyes surrounded by black pigmentation; ALE largest, oval; PME squared; PLE smallest, oval; ALE separated by their radius; ALE-PLE touching; PLE-PME touching; PME touching. ***Abdomen***: Oval; whitish covered dorsally with long dark setae; epigastric and postepigastric scuta light orange, well sclerotized (Fig. 26). ***Legs***: Femora with basal half whitish, apical half, light yellow; other leg segments light yellow; tibia I with four pairs of ventral spines, metatarsus I with three pairs of ventral spines; leg formula 4123; all palpal segments with strong spines. ***Genitalia***: Epigynal region not protruding, with crcifix-shaped structure visible through the scutum and the epigastric furrow (Fig. 26). Internal genitalia with triangular anterior receptaculum, projecting posteriorly; posterior receptaculum elongated oval, with large pore field; wing-like projections arched (Fig. 27).

**Male**: Unknown.

###### Other material examined.

None.

###### Distribution.

Dominican Republic, La Vega Province (Fig. 40).

##### Heteroonops solanllycarreroae

Taxon classificationAnimaliaAraneaeOonopidae

Dupérré sp. nov.

3D640485-4E00-5372-BD6C-39302506D195

http://zoobank.org/F190F990-F3D6-4881-B509-382DE2BEA50C

Figs 28 , 29 , 40

###### Type material.

Female holotype from Dominican Republic, Duarte Province, Occidental, San Rafael de Yuma, Loma Quita Espuela, 19.35504N, 70.111W, 200 m, 14.vi.2012, team CarBio (NMNH, USNMENT 01747012).

###### Etymology.

The specific epithet is a noun in apposition honoring local arachnologist and CarBio collaborator Solanlly Carrrero.

###### Diagnosis.

Females are diagnosed from all species by their posteriorly protruding epigastric scutum and their oval posterior receptaculum with folded bag-like extension (Fig. 29).

###### Description.

**Figures 28, 29.**
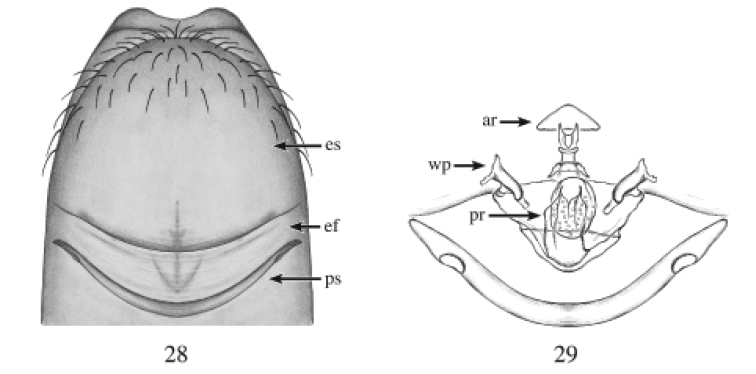
*Heteroonops solanllycarreroae* sp. nov.. Female. **28** Epigynal region, ventral view **29** internal genitalia, dorsal view.

**Female (holotype).** Total length: 1.37; carapace length: 0.61; carapace width: 0.42. ***Cephalothorax***: Carapace ovoid; shiny, whitish; pars cephalica flat. Sternum whitish; longer than wide; covered entirely with long dark setae. Endites whitish, not modified; labium whitish. Clypeus vertical; short (1/2× radius of ALE). Chelicerae whitish; promargin and retromargin without teeth; fangs normal, 1/3 length of chelicerae. ***Eyes***: Six eyes surrounded by black pigmentation; ALE largest, oval; PME squared; PLE smallest, oval; ALE separated by their radius; ALE-PLE touching; PLE-PME touching; PME touching. ***Abdomen***: Oval; light gray covered dorsally with long dark setae; epigastric scutum protruding, postepigastric scutum thin; scuta light yellow, not well sclerotized (Fig. 28). ***Legs***: Whitish; tibia I with three pairs of ventral spines, metatarsus I with two pairs of ventral spines; leg formula 4123; all palpal segments with strong spines. ***Genitalia***: Epigynal region protruding ventrally (not visible on image) with anchor-shaped structure visible through the epigastric scutum and epigastric furrow (Fig. 28). Internal genitalia with hat-shaped anterior receptaculum; posterior receptaculum oval with small pore field region and folded bag-like extension; wing-like projections anvil-shaped (Fig. 29).

**Male**: Unknown.

###### Other material examined.

None.

###### Distribution.

Dominican Republic, La Duarte Province (Fig. 40).

##### Heteroonops constanza

Taxon classificationAnimaliaAraneaeOonopidae

Dupérré sp. nov.

A0EE1E54-3663-5884-AA76-1614129215C8

http://zoobank.org/C1FAE1A8-EA65-4320-8419-A24E63086580

**Figures 30–33.**
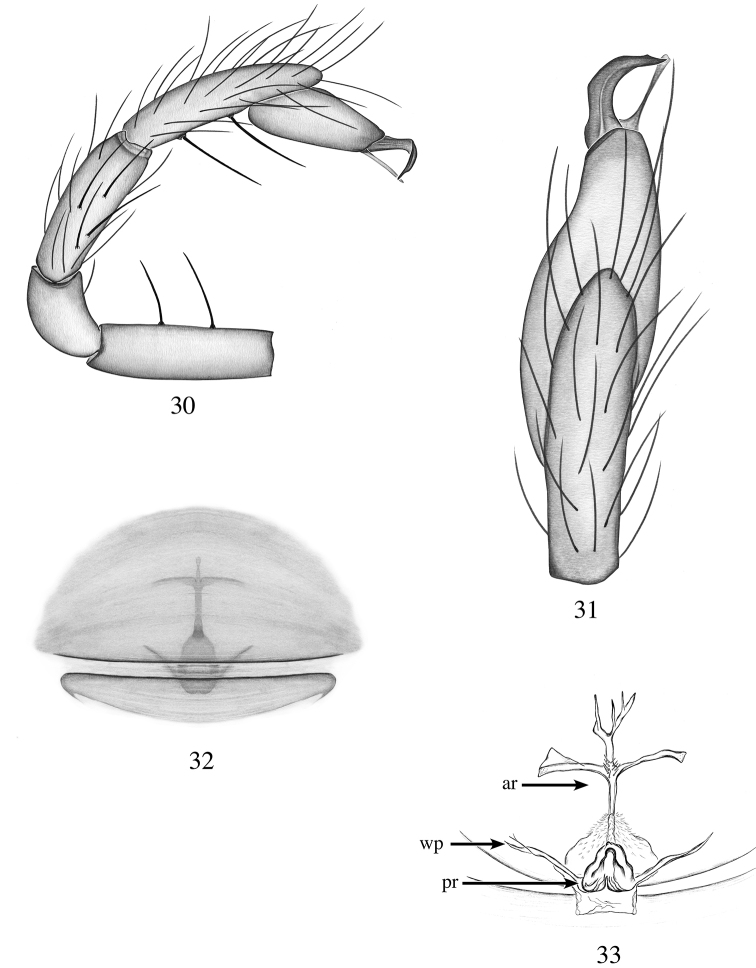
*Heteroonops constanza* sp. nov. Male (**30, 31**), female (**32, 33**). **30** Palp, prolateral view **31** palp, apical view **32** epigynal region, ventral view **33** internal genitalia, dorsal view.

Figs 30–33 , 39 , 40

###### Type material.

Male holotype from Dominican Republic, La Vega Province, Constanza, Valle Nuevo National Park, ‘Jurassic Park’, 18.688N, 70.596W, 2100 m, 26.vi.2012, team CarBio (NMNH, USNMENT 01747013). Two female paratypes (USNMENT 01747014), same data.

###### Etymology.

The specific name is noun in apposition taken from the type locality, Constanza Province, Dominican Republic.

###### Diagnosis.

Both males and females closely resemble *H. castelloides* Platnick & Dupérré, 2009; males are distinguished by the narrow, elongated palpal bulb and palpal tibia 2× longer than patellae (Fig. 30), ovoid in the later species, and palpal tibia 1.5× longer than patellae (Platnick and Dupérré 2009, fig. 242); females are distinguished by their anterior recepetaculum with four branches (Fig. 33), five in *H. castelloides* (Platnick and Dupérré 2009, fig. 259).

###### Description.

**Male (holotype)**: Total length: 1.79; carapace length: 0.86; carapace width: 0.72. ***Cephalothorax***: Carapace ovoid; shiny, pale yellow; pars cephalica slightly elevated. Sternum pale yellow; longer than wide; covered entirely with long dark setae. Endites pale yellow, with small apical projection (Fig. 39); labium light yellow. Clypeus sligthly protruding; short (1/2× radius of ALE). Chelicerae yellow; promargin and retromargin without teeth; fangs normal, 1/3 length of chelicerae. ***Eyes***: Six eyes surrounded by black pigmentation; ALE largest, oval; PME rounded; PLE smallest, oval; ALE separated by their radius; ALE-PLE touching; PLE-PME touching; PME touching. ***Abdomen***: Oval; beige covered dorsally with long setae; epigastric and postepigastric scuta inconspicuous. ***Legs***: Legs missing. ***Genitalia***: Palpal segments pale yellow; palpal bulb whitish. Palpal femora, tibia and tarsus with spines prolaterally (Fig. 30). Palpal bulb elongated (Fig. 30); embolus strongly bent, pointed apically; conductor long and thin reaching the tip of the embolus (Fig. 31).

**Female (paratype)**: Total length: 2.09; carapace length: 0.85; carapace width: 0.72. ***Cephalothorax***: Carapace ovoid; shiny, yellow; pars cephalica flat. Sternum and labium light yellow. Chelicerae and endites light yellow, not modified. ***Eyes***: as in male. ***Abdomen***: Oval, light beige; epigastric and postepigastric scuta pale yellow, not well sclerotized (Fig. 32). ***Legs***: Legs missing; all palpal segments with strong spines. ***Genitalia***: Epigynal region not protruding, with tree-shaped structures slightly visible through the epigastric scutum (Fig. 32). Internal genitalia with anterior receptaculum elongated with four main branches; posterior receptaculum triangular well sclerotized; wing-like projections elongated and narrow (Fig. 33).

###### Other material examined.

None.

**Figures 34–39.**
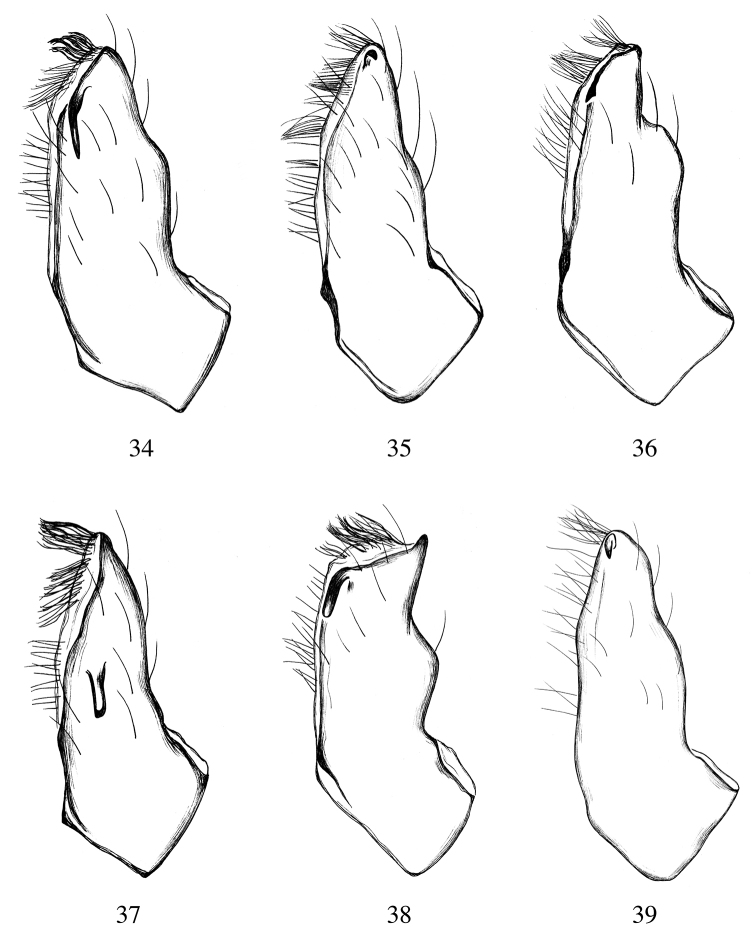
Male endites, ventral view. **34***Heteroonops scapula* sp. nov. **35***Heteroonops jurassicus* sp. nov. **36***Heteroonops aylinalegreae* sp. nov. **37***Heteroonops verruca* sp. nov. **38***Heteroonops renebarbai* sp. nov. **39***Heteroonops constanza* sp. nov.

###### Distribution.

**Figure 40.**
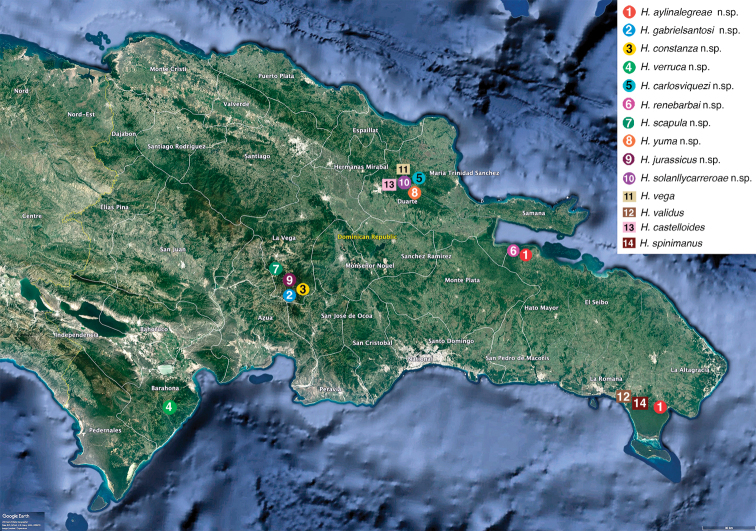
Distribution map of all *Heteroonops* species found in Hispaniola.

Dominican Republic, La Vega Province (Fig. 40).

### New records

#### Heteroonops spinimanus

Taxon classificationAnimaliaAraneaeOonopidae

(Simon, 1891)

6B0D1243-AEAE-59EE-BB2F-93AB03C87058

Fig. 40

##### Material examined.

Dominican Republic, La Alta Gracia Province, Occidental, San Rafael de Yuma, del Este National Park, beach Trail to Cueva del Puente, 18.32902N, 68.80995W, 0 m, 5.vi.2012, team CarBio,1♀ (UVM).

#### Heteroonops castelloides

Taxon classificationAnimaliaAraneaeOonopidae

Platnick & Dupérré, 2009

10301FC4-03CD-5822-A47F-E395DF228073

Fig. 40

##### Material examined.

Dominican Republic, La Duarte Province, Occidental, San Rafael de Yuma, Loma Quita Espuela, 19.35504N, 70.111W, 200 m, 14.vi.2012, team CarBio, 1♂ (UVM).

#### Heteroonops validus

Taxon classificationAnimaliaAraneaeOonopidae

(Bryant, 1948)

A0103482-1EB1-59F7-9CD3-F2A7D4A6D418

Fig. 40

##### Material examined.

Dominican Republic, La Alta Gracia Province, Occidental, San Rafael de Yuma, del Este National Park, Cueva del Puente, 18.3816N, 68.8017W, 25 m, 6.vi.2012, team CarBio, 3♂4♀ (UVM).

#### Heteroonops vega

Taxon classificationAnimaliaAraneaeOonopidae

Platnick & Dupérré, 2009

6A3BF836-79F3-589A-8E15-409428D50394

Fig. 40

##### Material examined.

Dominican Republic, La Duarte Province, Occidental, San Rafael de Yuma, Loma Quita Espuela, 19.35504N, 70.111W, 200m, 14.vi.2012, team CarBio, 1♂ (UVM).

## Discussion

Observed patterns in our data are consistent with a high probability that our sampling has only detected a small subset of the *Heteroonops* diversity in Hispaniola. First, we found a total of 66 individuals distributed in 14 *Heteroonops* species, 10 of which were new, from only eight sampling sites. At a single site in Loma Quita (200 m) we found five species including three that are new (*H. yuma*, *H. carlosviquezi*, *H. solanllycarreroae*) and two that represent new records (*H. vega*, *H. castelloides*). Similarly, we found three new species in one locality in a high elevation forest (2100 m) in the Cordillera Central Parque National Valle Nuevo (*H. constanza*, *H. gabrielsantosi*, *H. jurassicus*). Moreover, a fourth new species *H. scapula*, was discovered in the same park at higher elevation (2983 m). Taxa from both of these localities are phylogenetically widespread reflecting an old most recent common ancestor and high levels of subsequent diversification (Fig. 1)). This contrasts with patterns seen in more dispersive Caribbean spiders that rarely have more than a single species of a given genus in one locality (e.g., Dziki et al. 2015, Agnarsson et al. 2018, Čandek et al. 2019, Tong et al. 2019)

Despite patterns consistent with high local diversity, there is evidence that some *Heteroonops* species are wide ranging. Two taxa that represent new records were collected far from their type localities in the Cordillera Central, *H. castelloides*, and *H. validus.* Interestingly both of these species have been collected in flight intercept traps (Platnick and Dupérré 2009) suggesting the potential for aerial dispersal. Additionally, one species described here, *H. aylinalegreae*, was collected in two separate low elevation localities on the northern and southern sides of Eastern Hispaniola. While it seems that some members of this genus are capable of widespread dispersal, most notably the type species, the high levels of diversity in the Dominican Republic suggest an old presence and much speciation within West Indies, consistent with biologies that are not typically dispersal prone.

## Supplementary Material

XML Treatment for Heteroonops

XML Treatment for Heteroonops scapula

XML Treatment for Heteroonops jurassicus

XML Treatment for Heteroonops aylinalegreae

XML Treatment for Heteroonops verruca

XML Treatment for Heteroonops renebarbai

XML Treatment for Heteroonops yuma

XML Treatment for Heteroonops carlosviquezi

XML Treatment for Heteroonops gabrielsantosi

XML Treatment for Heteroonops solanllycarreroae

XML Treatment for Heteroonops constanza

XML Treatment for Heteroonops spinimanus

XML Treatment for Heteroonops castelloides

XML Treatment for Heteroonops validus

XML Treatment for Heteroonops vega

## Supplementary materials

Supplementary material 1 Phylogeny in Figure 1 with full taxon labeling Data type Tree topology inferred using Bayesian analyses of mtDNA with each terminal taxon labeled Explanation note This tree is the same phylogeny as in Figure 1, however each terminal taxon is labeled with details that links that taxon with information in Table 1. Each label includes the name of the species, the sex (f/m), whether the specimen represents a holotype (h) or paratype (p), and a secondary label used to track the specimen through our analysis process. These labels connect the terminals with locality information and GenBank accession numbers detailed in Table 1. Values at nodes indicate posterior probabilities (and correspond to branch width). https://binary.pensoft.net/file/446507This dataset is made available under the Open Database License (http://opendatacommons.org/licenses/odbl/1.0/). The Open Database License (ODbL) is a license agreement intended to allow users to freely share, modify, and use this Dataset while maintaining this same freedom for others, provided that the original source and author(s) are credited.Nadine Dupérré, Charlotte Francisco, Ella Santana-Propper, Ingi Agnarsson, Greta J. Binford
