# Supplementary figures and images for: Heteroonops (Araneae, Oonopidae) spiders from Hispaniola: the discovery of ten new species
Source: Zookeys. 2020 Aug 27;964:1–30. doi: 10.3897/zookeys.964.51554 (PMC7471135; doi:10.3897/zookeys.964.51554)

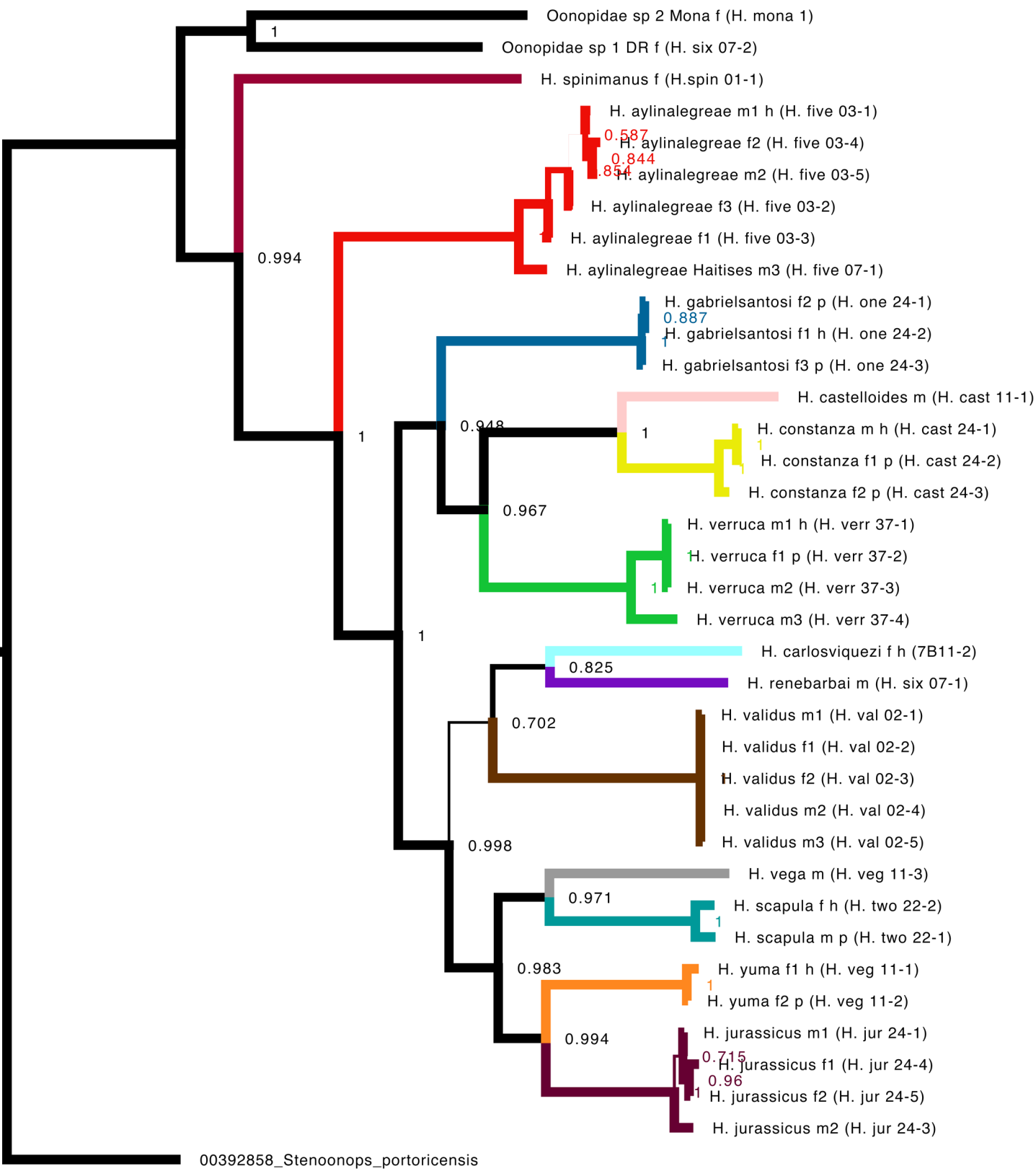

00392858\_Stenoonops\_portoricensis

0.05

Supplement: Supplementary material 1 — Phylogeny in Figure 1 with full taxon labeling [file zookeys-964-001-s001.pdf]
